# Supplementary material for: Modeling glioblastoma heterogeneity as a dynamic network of cell states
Source: Mol Syst Biol. 2021 Sep 16;17(9):e10105. doi: 10.15252/msb.202010105 (PMC8444284; doi:10.15252/msb.202010105)
Supplement: Supplementary file 5 — Source Data for Figure 3 [file MSB-17-e10105-s001.zip › Figure3A_sourcedata/GSEA_3065/nosigdb_state3.GseaPreranked.1622718426028/RICHARDS_VERHAAK_CANCERCELL_2010_PRONEURAL.html]

Details for gene set RICHARDS\_VERHAAK\_CANCERCELL\_2010\_PRONEURAL[GSEA]

|  || Dataset | state3 |
| Phenotype | NoPhenotypeAvailable |
| Upregulated in class | na\_pos |
| GeneSet | RICHARDS\_VERHAAK\_CANCERCELL\_2010\_PRONEURAL |
| Enrichment Score (ES) | 0.47036722 |
| Normalized Enrichment Score (NES) | 1.8755211 |
| Nominal p-value | 0.0 |
| FDR q-value | 0.0027310639 |
| FWER p-Value | 0.025 |
Table: GSEA Results Summary

  

Fig 1: Enrichment plot: RICHARDS\_VERHAAK\_CANCERCELL\_2010\_PRONEURAL      
 Profile of the Running ES Score & Positions of GeneSet Members on the Rank Ordered List

  

| PROBE | GENE SYMBOL | GENE\_TITLE | RANK IN GENE LIST | RANK METRIC SCORE | RUNNING ES | CORE ENRICHMENT || 1 | BCAN |  |  | 0 | 0.883 | 0.0642 | Yes |
| 2 | GPM6A |  |  | 37 | 0.460 | 0.0935 | Yes |
| 3 | GRIA2 |  |  | 38 | 0.460 | 0.1269 | Yes |
| 4 | ALCAM |  |  | 45 | 0.440 | 0.1582 | Yes |
| 5 | NCAM1 |  |  | 52 | 0.430 | 0.1887 | Yes |
| 6 | MMP16 |  |  | 94 | 0.351 | 0.2095 | Yes |
| 7 | SOX11 |  |  | 117 | 0.325 | 0.2305 | Yes |
| 8 | DBN1 |  |  | 159 | 0.306 | 0.2480 | Yes |
| 9 | CHD7 |  |  | 172 | 0.298 | 0.2683 | Yes |
| 10 | TTYH1 |  |  | 183 | 0.293 | 0.2884 | Yes |
| 11 | MYO10 |  |  | 206 | 0.280 | 0.3063 | Yes |
| 12 | ADGRL3 |  |  | 290 | 0.247 | 0.3146 | Yes |
| 13 | MAP2 |  |  | 344 | 0.231 | 0.3253 | Yes |
| 14 | ZEB2 |  |  | 401 | 0.218 | 0.3346 | Yes |
| 15 | LRP6 |  |  | 458 | 0.207 | 0.3432 | Yes |
| 16 | ATAD5 |  |  | 470 | 0.204 | 0.3567 | Yes |
| 17 | TOP2B |  |  | 508 | 0.198 | 0.3668 | Yes |
| 18 | DPP6 |  |  | 534 | 0.194 | 0.3781 | Yes |
| 19 | NLGN3 |  |  | 558 | 0.191 | 0.3893 | Yes |
| 20 | MTSS1 |  |  | 576 | 0.189 | 0.4010 | Yes |
| 21 | CASK |  |  | 643 | 0.180 | 0.4065 | Yes |
| 22 | CSPG5 |  |  | 716 | 0.168 | 0.4103 | Yes |
| 23 | P2RX7 |  |  | 723 | 0.167 | 0.4218 | Yes |
| 24 | SEC61A2 |  |  | 739 | 0.165 | 0.4321 | Yes |
| 25 | DNM3 |  |  | 783 | 0.159 | 0.4387 | Yes |
| 26 | PODXL2 |  |  | 806 | 0.157 | 0.4476 | Yes |
| 27 | PHLPP1 |  |  | 819 | 0.156 | 0.4575 | Yes |
| 28 | RUFY3 |  |  | 848 | 0.153 | 0.4653 | Yes |
| 29 | ERBB3 |  |  | 899 | 0.149 | 0.4704 | Yes |
| 30 | DLL3 |  |  | 999 | 0.138 | 0.4690 | No |
| 31 | MCM10 |  |  | 1197 | 0.122 | 0.4550 | No |
| 32 | CLASP2 |  |  | 1350 | 0.112 | 0.4456 | No |
| 33 | SRGAP3 |  |  | 1353 | 0.111 | 0.4535 | No |
| 34 | ARHGEF9 |  |  | 1402 | 0.108 | 0.4558 | No |
| 35 | TOPBP1 |  |  | 1474 | 0.105 | 0.4552 | No |
| 36 | EPB41 |  |  | 1504 | 0.103 | 0.4593 | No |
| 37 | BCOR |  |  | 1634 | 0.095 | 0.4513 | No |
| 38 | BCL7A |  |  | 1677 | 0.093 | 0.4532 | No |
| 39 | C1orf61 |  |  | 1703 | 0.091 | 0.4569 | No |
| 40 | CAMSAP2 |  |  | 1704 | 0.091 | 0.4636 | No |
| 41 | OLIG2 |  |  | 1742 | 0.089 | 0.4658 | No |
| 42 | ARHGAP33 |  |  | 2065 | 0.073 | 0.4339 | No |
| 43 | RAD21 |  |  | 2150 | 0.070 | 0.4293 | No |
| 44 | TSPAN3 |  |  | 2828 | 0.046 | 0.3543 | No |
| 45 | ZNF248 |  |  | 2876 | 0.045 | 0.3522 | No |
| 46 | RBPJ |  |  | 2897 | 0.044 | 0.3531 | No |
| 47 | REEP1 |  |  | 2960 | 0.042 | 0.3490 | No |
| 48 | RNFT2 |  |  | 2967 | 0.042 | 0.3513 | No |
| 49 | ABAT |  |  | 3059 | 0.040 | 0.3437 | No |
| 50 | PAK3 |  |  | 3132 | 0.038 | 0.3381 | No |
| 51 | FHOD3 |  |  | 3226 | 0.035 | 0.3299 | No |
| 52 | MAPT |  |  | 3333 | 0.032 | 0.3200 | No |
| 53 | CDC7 |  |  | 3339 | 0.032 | 0.3217 | No |
| 54 | FBXO21 |  |  | 3474 | 0.029 | 0.3083 | No |
| 55 | KDM1A |  |  | 3485 | 0.028 | 0.3092 | No |
| 56 | E2F3 |  |  | 3553 | 0.027 | 0.3034 | No |
| 57 | GSK3B |  |  | 3606 | 0.025 | 0.2993 | No |
| 58 | TMCC1 |  |  | 3614 | 0.025 | 0.3003 | No |
| 59 | ZNF821 |  |  | 3808 | 0.020 | 0.2794 | No |
| 60 | DCAF7 |  |  | 3898 | 0.018 | 0.2704 | No |
| 61 | CDC25A |  |  | 4065 | 0.013 | 0.2522 | No |
| 62 | VEZF1 |  |  | 4169 | 0.011 | 0.2410 | No |
| 63 | DPYSL4 |  |  | 4376 | 0.005 | 0.2176 | No |
| 64 | HMGB3 |  |  | 4445 | 0.003 | 0.2099 | No |
| 65 | CSNK1E |  |  | 4623 | -0.002 | 0.1896 | No |
| 66 | KAT7 |  |  | 4632 | -0.002 | 0.1888 | No |
| 67 | ZNF184 |  |  | 4776 | -0.005 | 0.1727 | No |
| 68 | MMP15 |  |  | 4903 | -0.008 | 0.1587 | No |
| 69 | YPEL1 |  |  | 4935 | -0.009 | 0.1558 | No |
| 70 | AMOTL2 |  |  | 5031 | -0.012 | 0.1457 | No |
| 71 | MVB12B |  |  | 5074 | -0.013 | 0.1417 | No |
| 72 | FERMT1 |  |  | 5185 | -0.015 | 0.1301 | No |
| 73 | RALGPS2 |  |  | 5240 | -0.017 | 0.1251 | No |
| 74 | GADD45G |  |  | 5583 | -0.027 | 0.0875 | No |
| 75 | SATB1 |  |  | 5608 | -0.027 | 0.0867 | No |
| 76 | GSTA4 |  |  | 5649 | -0.028 | 0.0842 | No |
| 77 | CDKN1B |  |  | 5783 | -0.032 | 0.0711 | No |
| 78 | CNTN1 |  |  | 5924 | -0.037 | 0.0576 | No |
| 79 | WASF1 |  |  | 6025 | -0.040 | 0.0490 | No |
| 80 | SLCO5A1 |  |  | 6176 | -0.046 | 0.0349 | No |
| 81 | PPM1D |  |  | 6214 | -0.047 | 0.0341 | No |
| 82 | DPF1 |  |  | 6224 | -0.047 | 0.0364 | No |
| 83 | CRMP1 |  |  | 6243 | -0.048 | 0.0379 | No |
| 84 | ATAT1 |  |  | 6334 | -0.051 | 0.0311 | No |
| 85 | TMSB15A |  |  | 6711 | -0.067 | -0.0075 | No |
| 86 | FXYD6 |  |  | 6822 | -0.072 | -0.0150 | No |
| 87 | HDAC2 |  |  | 6905 | -0.076 | -0.0189 | No |
| 88 | SEZ6L |  |  | 6920 | -0.077 | -0.0150 | No |
| 89 | ACTR1A |  |  | 7021 | -0.082 | -0.0206 | No |
| 90 | RAP2A |  |  | 7092 | -0.086 | -0.0224 | No |
| 91 | GNG4 |  |  | 7391 | -0.108 | -0.0490 | No |
| 92 | ICK |  |  | 7483 | -0.115 | -0.0511 | No |
| 93 | CKB |  |  | 7659 | -0.134 | -0.0616 | No |
| 94 | STMN1 |  |  | 7808 | -0.153 | -0.0676 | No |
| 95 | TOX3 |  |  | 7951 | -0.177 | -0.0712 | No |
| 96 | CBX1 |  |  | 7995 | -0.184 | -0.0627 | No |
| 97 | SOX4 |  |  | 8123 | -0.209 | -0.0622 | No |
| 98 | PAFAH1B3 |  |  | 8326 | -0.264 | -0.0663 | No |
| 99 | C1QL1 |  |  | 8390 | -0.290 | -0.0526 | No |
| 100 | MARCKSL1 |  |  | 8415 | -0.300 | -0.0335 | No |
| 101 | BEX1 |  |  | 8450 | -0.314 | -0.0146 | No |
| 102 | MLLT11 |  |  | 8489 | -0.335 | 0.0054 | No |
| 103 | PFN2 |  |  | 8503 | -0.342 | 0.0287 | No |
Table: GSEA details [plain text format]

  

Fig 2: RICHARDS\_VERHAAK\_CANCERCELL\_2010\_PRONEURAL: Random ES distribution      
 Gene set null distribution of ES for **RICHARDS\_VERHAAK\_CANCERCELL\_2010\_PRONEURAL**
